# Somatic Mutations and Genetic Variants of NOTCH1 in Head and Neck Squamous Cell Carcinoma Occurrence and Development

**DOI:** 10.1038/srep24014

**Published:** 2016-04-01

**Authors:** Yu-Fan Liu, Shang-Lun Chiang, Chien-Yu Lin, Jan-Gowth Chang, Chia-Min Chung, Albert Min-Shan Ko, You-Zhe Lin, Chien-Hung Lee, Ka-Wo Lee, Mu-Kuan Chen, Chun-Hung Hua, Ming-Hsui Tsai, Yuan-Chien Chen, Ying-Chin Ko

**Affiliations:** 1 Department of Biomedical Sciences, College of Medicine Sciences and Technology, Chung Shan Medical University, Taichung, Taiwan; 2 Division of Allergy, Department of Pediatrics, Chung-Shan Medical University Hospital, Taichung, Taiwan; 3 Environment-Omics-Diseases Research Centre, China Medical University Hospital, Taichung, Taiwan; 4 Department of Health Risk Management, College of Public Health, China Medical University, Taichung, Taiwan; 5 Department of Laboratory Medicine, China Medical University Hospital, Taichung, Taiwan; 6 Department of Evolutionary Genetics, Max Planck Institute for Evolutionary Anthropology, Leipzig D-04103, Germany; 7 Department of Public Health, College of Health Science, Kaohsiung Medical University, Taiwan; 8 Department of Otolaryngology, Kaohsiung Medical University Hospital, Kaohsiung, Taiwan; 9 Oral Cancer Center, Changhua Christian Hospital, Changhua, Taiwan; 10 Department of Otorhinolaryngology, China Medical University Hospital, Taichung, Taiwan; 11 Department of Dentistry, China Medical University Hospital, Taichung, Taiwan; 12 Graduate Institute of Clinical Medical Science, China Medical University, Taichung, Taiwan

## Abstract

A number of genetic variants have been associated with cancer occurrence, however it may be the acquired somatic mutations (SMs) that drive cancer development. This study investigates the potential SMs and related genetic variants associated with the occurrence and development of head and neck squamous cell carcinoma (HNSCC). We identified several SMs in NOTCH1 from whole-exome sequencing and validated them in a 13-year cohort of 128 HNSCC patients using a high-resolution melting analysis and resequencing. Patients who have NOTCH1 SMs show higher 5-year relapse-free recurrence (*P* = 0.0013) and lower survival proportion (*P* = 0.0447) when the risk-associated SMs were analysed by Cox proportional hazard models. Interestingly, the NOTCH1 gene rs139994842 that shares linkage with SMs is associated with HNSCC risk (*OR* = 3.46), increasing when SMs in NOTCH1 are involved (*OR* = 7.74), and furthermore when there are SMs in conjunction to betel quid chewing (*OR* = 32.11), which is a related independent environmental risk factor after adjusting for substances use (alcohol, betel quid, cigarettes) and age. The findings indicate that betel quid chewing is highly associated with NOTCH1 SMs (especially with changes in EGF-like domains), and that rs139994842 may potentially serve as an early predictive and prognostic biomarker for the occurrence and development of HNSCC.

Head and neck squamous cell carcinoma (HNSCC), involving the lip, oral cavity, pharynx and larynx, is one of the most common cancers with 599,000 new cases and 325,000 cancer deaths worldwide in 2012^1^. The GLOBOCAN series of International Agency for Research on Cancer estimates the age-standardized incidence is highest in Taiwan, followed by Hungary, Belgium, France, Germany, parts of India, Melanesia, and little has changed since 2008–2012^1^,^2^.

Although correlations between genetic variants and HNSCC have been made, few single nucleotide polymorphisms (SNPs) are able to be replicated among different populations^3^. Thus, attention has turned to the exome high-throughput sequencing, which is a part of the Cancer Genome Atlas that has shown promising genes associated with the initiation and progression of cancer^4^. Whole-exome sequencing (WES) studies have implicated NOTCH1 in about 15–19% of HNSCC occurrence^5^,^6^.

The NOTCH1 protein is a single-pass transmembrane receptor known to exist in a wide range of tissues and organisms^7^. A major part of NOTCH1 is its extracellular region, comprising of 36 EGF-like domains that contain Ca^2+^-binding consensus sequence. The inactivation of NOTCH1 has been linked to squamous cell differentiation and HNSCC^8^, and NOTCH1 regulation of squamous epithelium differentiation is also suggested by studies using cultured cervical and oesophageal keratinocytes^9^,^10^. In spite of NOTCH1 is proposed to be an oncogene or tumour suppressor gene in human cancer development^11^, in the instance of HNSCC^4^,^5^,^6^, it may be a tumour suppressor gene similar to cutaneous squamous cell carcinomas.

Because Taiwan, India, and Melanesia are betel chewing prevalent areas, whereas Central and Eastern European countries, such as France, Germany and Hungary are heavy alcohol drinking areas^12^,^13^,^14^, as well as epidemiological studies have linked HNSCC with the use of alcohol, betel quid, cigarettes and various genes^3^,^13^,^15^ and tobacco has been studied as a contributing factor to somatic mutations (SMs) of NOTCH1 in HNSCC^5^,^6^; this study investigates the potential SMs and related genetic variants and environmental (or substances use) risk factors that are associated with the occurrence and development of HNSCC. The effects of SMs are analysed in terms of recurrence and survival from HNSCC.

## Results

### Patient characteristics in SM Validation

Table 1 shows the clinical characteristics of the 128 HNSCC patients used for validation of SMs. Among these, 23 (18%) have NOTCH1 SMs. The mean ages of patients with and without NOTCH1 SMs were 52.8 and 51.3 years old. No significance difference was observed for the cancer sites, stages, and adjuvant therapies (radiotherapy and chemotherapy). However, significant differences were observed for the malignant tumour recurrence (*P* = 0.02) and fatality rate (borderline *P* = 0.06).

### Structural characteristics of NOTCH1 SMs

**Figure 1:**
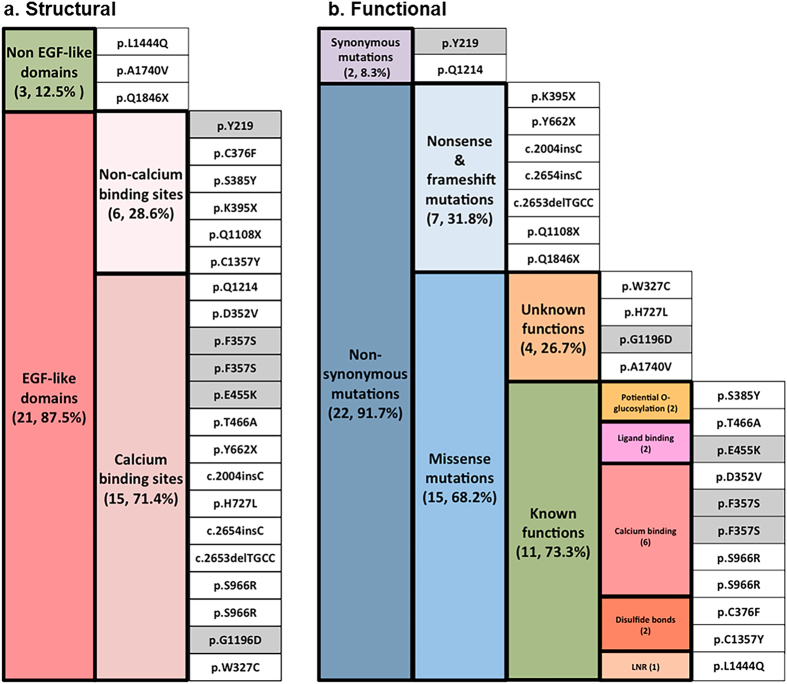
Characteristics of 24 SMs in NOTCH1 coding region from 23 HNSCC patients (n, %). (**a**) Structural and (**b**) Functional catalogues. Grey blocks revealed the SMs were annotated in the COSMIC database (v.73), while the white blocks indicate novel SMs investigated in this study. SMs were arranged to emphasize mutual exclusivity. The types of SMs were indicated in different colours.

**Figure 2:**
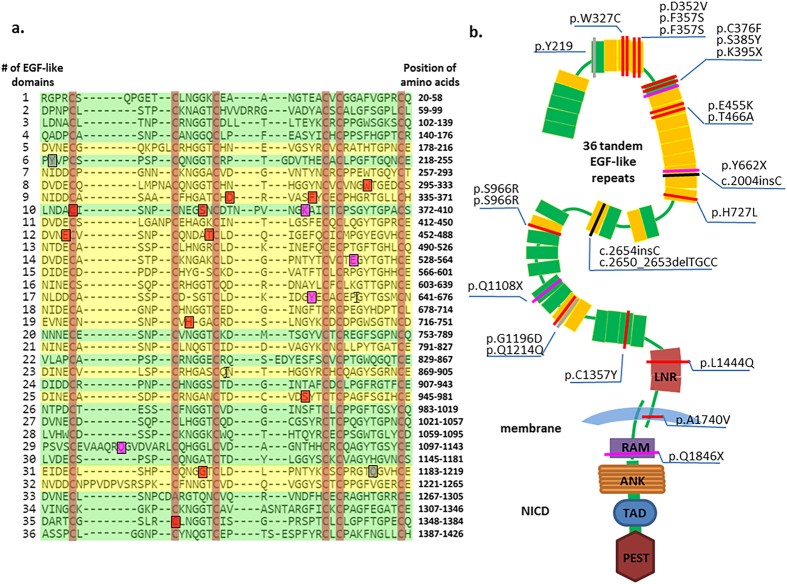
Somatic mutations distributed across the region of NOTCH1 receptor in 23 HNSCC patients. (**a**) An alignment of 36 tandem EGF-like domains of human NOTCH1 extracted from the UniProt protein database and generated by Align tools using the Clustal Omega programme according to the EGF-like repeats consensus. Each line represents a conserved EGF-like domain, consensus site for Ca^2+^ dependent binding (shaded yellow) and non-Ca^2+^ binding (shaded green) among 36 EGF-like repeats in the extracellular domains of a fold “triple-stranded” structure model. Red highlighting indicates six conserved Cysteine residues of the EGF-like domain to form consensus disulfide bonds. Blue and green boxes show the somatic mutation identified from this study of 124 HNSCC patients. Grey, red and purple shading in boxes show synonymous, missense and nonsense somatic mutations at the EGF-like domain, respectively. The symbol of “I” indicates the frameshift mutation. (**b**) Schematic diagram of the domain organization of the human NOTCH1 gene generated by the SMART database including 36 tandem EGF-like repeats (colour yellow and green indicate the Ca^2+^-dependent and non- Ca^2+^ binding domain, respectively; rectangle) and 3 Lin-12/Notch repeats (LNR; colour green; rectangle), 2 hetero- dimerization domain (HD; Colour grey; rectangle) determined as negative regulatory regions. A short transmembrane segment (TM; colour blue; arc). The Notch intracellular domain (NICD) contains the recombination signal-binding protein 1 for J (RBP-J) association molecule (RAM; colour red; rectangle), Ankyrin repeats (ANK; colour orange; rectangle), transcriptional activation domain (TAD; colour deep blue; rectangle) and proline, glutamic acid, serine/threonine-rich motif (PEST; colour brown; rectangle). Each colour bar represents a NOTCH1 somatic mutation in an HNSCC individual, of the class of mutation type indicated the same colour as (**a**).

Twenty-four SMs distributed across 34 exons of the *NOTCH1* gene were found in the cancerous tissues of 23 HNSCC patients (Table S1). Twenty-one SMs (87.5%), including 15 (71.4%) at Ca^2+^ binding sites and 6 (28.6%) at non-Ca^2+^ binding sites, were located in EGF-like domains of the NOTCH1 extracellular region (Fig. 1a). The mutation category view showed 22 alternations comprising 19 point mutations, 1 single-base deletion and 2 mononucleotide insertions. Given the novelty of SMs, 4 SMs were found in the database of COSMIC v73, and 18 SMs were identified for the first time in this study. To elucidate the relationship between the NOTCH1 SMs and the functional diversity, the structural consequences of the respective SMs in proteins were assessed. Figure 2a presents the detailed positions of 19 SMs in EGF-like domains. Three NOTCH1 SMs were outside EGF-like domains, including 1 in the LNR region, 1 in the TM region and 1 in the RAM (Fig. 2b).

### *In silico* prediction of functional impact of NOTCH1 SMs

Functionally, 22 of the 24 SMs (91.7%) that was detected in 23 HNSCC patients were non-synonymous mutations, comprising 7 novel nonsense and frameshift SMs (31.8%) and 15 missense mutations (68.2%) (Fig. 1b). NOTCH1 is regarded as a tumour suppressor in HNSCC because these missense SMs within the domain frequently harboured potential protein inactivation or were located in domains that affected the conserved residues in the *NOTCH1* gene (Fig. 2b). Furthermore, these SMs have the potential to induce persistent NOTCH1 functional defects and to change the capacity of NOTCH1 in a manner that is indispensable for its interaction with ligands. The effects might be similar to those of NOTCH1 downregulation.

To quantify the extent to which the HNSCC phenotype can be explained by a destructive effect on protein structures or functions, these SMs are mapped onto the known 3D structure of the NOTCH1 protein (Fig. S4). Four missense SMs located in the Ca^2+^-binding EGF-like domain at three novel positions [c.1055A > T (p.D352V), c.1363G > A (p.E455K) and c.2898C > G (p.S966R)] and one known position [c.1070T > C (p.F357S)], as found in the COSMIC database, were predicted to influence the effectiveness of Ca^2+^-binding function (Fig. S4a). Another two novel missense SMs [c.1127G > T (p.C376F) and c.4070G > A (p.C1357Y)] were among the essential disulfide bonds of two stranded beta sheets between cysteine loops of the canonical EGF-like domain (Fig. 2a). Two SMs [c.1363G > A (p.E455K) and c.1396A > G (p.T466A) occurred in the ligand-binding region of NOTCH1. The T466A also formed part of the *O*-fucosylation consensus sequence within the domain that increase affinity for ligands Jagged 1 and DLL1^16^. Another novel point SM c.1154C > A (S385Y) was located within the conserved enzymatic *O*-fucosylation site by *O*-fucosyl-transferase in mammals (Fig. S4b).

### Association between different NOTCH1 SMs statuses and clinical parameters

**Figure 3:**
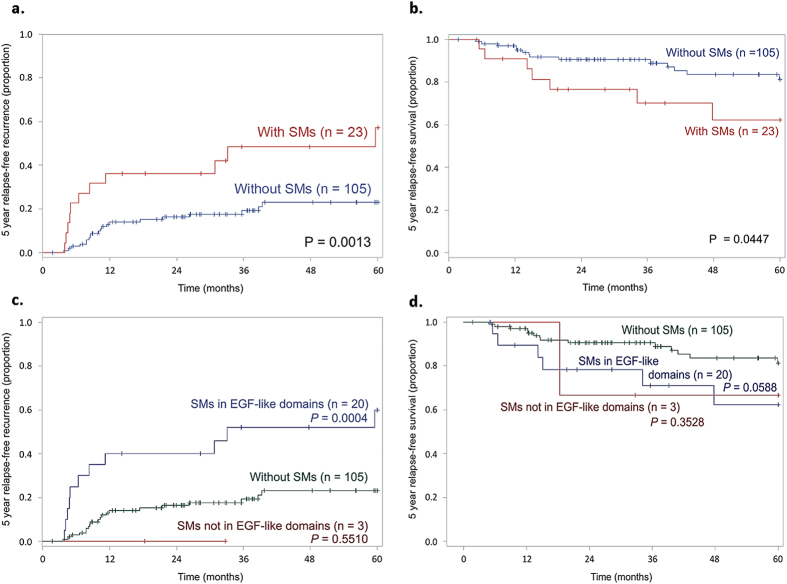
Kaplan-Meier estimates of 5-year relapse-free recurrence and survival proportion in 128 HNSCC patients. (**a**) Patients with SMs have significantly higher recurrence rate, (**b**) Patients with SMs have significantly lower survival rate, (**c**) Patients with SMs in EGF-like domain (representing majority of SMs found in this study) have significantly higher recurrence rate, (**d**) Patients with SMs in EGF-like domains may have significantly lower survival rate.

A recurrence of the malignant tumour was detected in 39 patients (30.5%); 22 of these 39 patients (17.2%) died of HNSCC during the 13-year follow-up. Patients who carried NOTCH1 SMs were at a higher risk of cancer recurrence [odds ratios (OR) = 4.5; 95% confidence interval (CI), 1.4–14.1; *P* = 0.01] and cancer death (OR = 5.8; 95% CI, 1.5–23.0; *P* = 0.01) than those who carried wild-types (Table 2). The Kaplan–Meier survival curves for patients with and without NOTCH1 SMs revealed significantly different 5-year relapse-free recurrence and survival curves (Fig. 3; *P* = 0.0013 and *P* = 0.0447, respectively). Multivariate regression analysis demonstrated that NOTCH1 SMs [hazard ratio (HR) = 3.2, *P* < 0.01) is an independent prognostic factor associated with 5-year disease-free recurrence for HNSCC patients; the HR increased to 5.2-fold (*P* < 0.01) after controlling for age at surgery, disease status (cancer site and stage), and adjuvant therapies (radiotherapy and chemotherapy) (also refer Table 2). Similar results were obtained for the 10-year disease-free survival analysis (Fig. S5). Moreover, after controlling for age at surgery, disease status and adjuvant therapies, NOTCH1 SMs (HR = 5.2, *P* < 0.01) were a prognostic factor for the 5-year disease-free survival of HNSCC patients.

### SMs-associated genetic polymorphism and environmental risk factors in a case-control study

The mean age of the patients with HNSCC and the controls were 53.8 years and 50.8 years old (Table S3). The proportions of males were 94.7% in the case group and 97.5% in the control group. Among the 282 cases, 67.4% were alcohol drinkers [controls: 29.1% (n = 282)], 82.3% were betel quid chewers [controls: 13.5% (n = 282)], and 86.2% were cigarette smokers [controls: 52.8% (n = 282)].

We found that, for example, 5 SMs (p.S385Y, p.E455K, p.T466A, p.S966R and p.Q1108X) were located in the EGF-like domains. Additionally, SNP rs139994842 showed a moderate correlation coefficient with these 5 NOTCH1 SMs (D’ = 1; *r* = 0.63; *P* = 0.0004) as well as significantly associated with increased risk of HNSCC (OR = 3.46; 95% CI, 1.11–10.84) (Table 3). The adjusted OR between HNSCC patients and HNSCC patients further identified with SMs in NOTCH1 were 3.46 (95% CI, 1.11–10.84) and 7.74 (95% CI, 1.70–35.31). No significant relationship between the group without SMs in NOTCH1 and the control groups was found (OR = 1.15; 95% CI, 0.24–5.50).

The use of alcohol, betel quid, and cigarettes were significantly associated with HNSCC. After adjusting for age and substance use, adjusted ORs in the betel chewing group increased from 22.45 (95% CI, 13.39–37.64) for HNSCC, to 31.55 (95% CI, 13.00–76.60) for NOTCH1 without SMs, and to 32.11 (95% CI, 10.41–99.05) for NOTCH1 with SMs. The adjusted OR also indicated an association between HNSCC and alcohol consumption in patients without SMs (OR = 5.71, 95% CI, 2.39–13.64; Table 3).

## Discussion

We observed a high fraction (68.2%) of HNSCC-related NOTCH1 SMs are missense mutations that locate in the functionally conserved residues within or close the extracellular region of ligand interaction. A lesser extent (31.8%) are nonsense and frameshift SMs that relate to truncated NOTCH1 proteins that lack C-terminal Notch intracellular domain (NICD) that may affect transactivation of target genes. A reduced NOTCH1 expression influences the terminal differentiation of squamous epithelium cells and forms immature epithelia, suggesting its essential role in maintaining the epithelial integrity^8^. An increased association with skin cancer risk^17^ from gamma-secretase inhibitors that target the signalling pathway downstream of NOTCH1 for Alzheimer’s disease has also been shown. The data are consistent with the NOTCH1 function as a tumour suppressor gene in HNSCC occurrence^4^.

According to our findings, the NOTCH1 SMs in patients were not only associated with higher risks of cancer recurrence and lower survival in 5-year (Fig. 3) and 10-year (Fig. S5) Kaplan Meier survival estimates but also had a significant predictive power in multivariate Cox regression for both cancer recurrence and death after controlling for patient- and hospital-confounders (Table 2). We further found that carriers of NOTCH1 genetic variant rs139994842 were associated with five SMs of NOTCH1 and could be used to predict risk of HNSCC. Since the SM generation is random, a biological reason remains to be investigated in future studies.

The HNSCC risk-associated rs139994842 is elevated further by betel quid (BQ) chewing, which is an independent risk factor^13^,^18^,^19^ that accounts for 79% of oral cancer and 18% of laryngeal cancer occurrence^20^. The typical BQ is a mixture of areca nut, betel leaf and slaked lime, and in some parts of the world, includes tobacco as an ingredient. BQ is evaluated to be a group 1 carcinogen to humans^21^ with an estimated 600 million users in the world^22^. A commercial formulation in Taiwan comprise of an areca nut, betel leaf (or inflorescences) and slaked lime. The preparation involving only areca nut or BQ containing tobacco is rarely consumed in Taiwan. The substance use associated with HNSCC in the Central and Eastern European countries may be heavy alcohol drinking^14^. We found that BQ chewing is significantly associated with HNSCC and NOTCH1 exome SMs, while alcohol drinking is associated with HNSCC and patients without NOTCH1 SMs (Table 3). Possibly, several NOTCH1 SMs increase the mutagenic effects of BQ, but not of alcohol. The effect of cigarette smoking was masked by that of betel chewing which had a stronger effect in this study.

In conclusion, our findings are consistent with previous reports of NOTCH1 SMs to associate with HNSCC^7^,^8^,^20^,^23^,^24^. Furthermore, we show that BQ chewing is strongly linked to the development of HNSCC through NOTCH1 SMs. These SMs are largely located to EGF-like domains that may functionally compromise and increase HNSCC recurrence and fatality, suggesting that NOTCH1 performs a tumour suppressive role in HNSCC. While rs139994842 relates to the germline, we show that it is possible to statistically serve as an early predictive and prognostic biomarker for the occurrence and development of HNSCC. This information can be used in prevention, surveillance of patients at risk, and early detection for reducing morbidity and mortality from HNSCC.

## Methods

### Patients and tissue specimens

Paired tissues (cancerous and normal marginal sections) were obtained from 3 male HNSCC patients at Kaohsiung Medical University Hospital (KMUH) for whole-exome SMs discovery. To validate these SMs, we recruited 128 male HNSCC patients (<6% of patients receiving adjuvant radiotherapy and/or chemotherapy before surgery) who have high quality paired tissue DNA between November 2000 and March 2012 (13 years follow-up) from China Medical University Hospital (CMUH). To investigate the association with substance use, 282 male patients diagnosed with HNSCC and 282 matched controls were recruited from KMUH for a case-control study. The three HNSCC cohorts are mutually independent; an overview is provided in Fig. S1.

Whole blood was obtained from volunteers with written informed consent. Information about social-demographic factors, anthropometric parameters, medical history, medications, and substance use (alcohol, betel quid (BQ), and cigarettes) were carefully recorded. Details regarding alcohol, BQ, cigarette use have included: types consumed, age at initial use, daily consumption, frequency of use, years of use, and achievement of abstinence^18^. The use of alcohol, BQ, and cigarettes were recorded in the newly diagnosed HNSCC patients at a first-time interview. An individual who has used alcohol, BQ, and cigarettes was defined as a drinker, chewer, and smoker. Genomic DNA was extracted from peripheral blood samples in case-control study using Puregene DNA isolation kit (Gentra Systems, Minneapolis, MN). This study was approved by the institutional review boards of KMUH and CMUH, committee on human subjects and biospecimen unitization committee (KMUH-DC-101-0402 and CMUH-HBB102-007). All methods were carried out in accordance with the approved guidelines.

### SMs screening and validation

A whole-exome sequencing (WES) discovery platform screened for candidate SMs from the paired tissues DNA of 3 HNSCC patients. DNA quantitation was determined from Qubit Fluorometer (Thermo Fisher Scientific). The whole-exome regions were captured using SureSelect Target Enrichment System (Agilent). A total of 6.5 gigabases sequence data was generated from next generation sequencing (NGS) using Solexa Hiseq 2000 sequencing system (Illumina). The NGS procedures^25^ of data cleaning, alignment, variant calling, and annotations are described in Fig. S1.

The raw WES data that were generated by massively parallel sequencing platform required 80-fold enrichment for all prepared cancer-normal pair libraries. Reads that contained sequencing adaptors and low-quality reads with more than five unknown bases were removed. The high-quality reads were aligned to UCSC human reference genome (hg19) using two software tools, BWA^26^ and Bowtie2^27^. To identify potential variants, local realignments of BWA-aligned reads were conducted using a genome analysis toolkit (TCGA)^28^. The raw lists of potential variants were then annotated, individually analysed, validated, and converted into prevalent types of variant call formats using VCFtools^29^. Potential SMs were detected in the matched non-tumorous HNSCC samples and the loci in exon regions. Another strategy was to directly compare sequences from the tumour and matched normal tissue during discovery or validation. Two applications were used to reveal specific mutations of the tumour: MuTect^30^ and SomaticSniper^31^. A Bayesian comparison was then performed to detect SMs with various allele fractions. The ANNOVAR^32^ tool was used to annotate the functions of these variants, to elucidate their effects on genes, and to obtain other information about known variants that were reported in the 1000 Genome Project^33^ and dbSNP databases^34^. Suitable specific primers were designed to verify potential SMs using Sanger sequencing, and the candidate SMs were surveyed by the Mutation Surveyor software (Fig. S2; version 4.0.6, Softgenetics, State College, PA)^35^. The novelty of SMs was assessed using the Catalogue of Somatic Mutations in Cancers (COSMIC v.73)^36^.

### Detection and validation of NOTCH1 SMs with high-resolution melting

All hotspots of *NOTCH1* exome SMs and genetic variants were identified from 128 male HNSCC patients using a high-resolution melting (HRM) analysis^37^ and verified by Sanger resequencing (also refer Fig. S3). PCR reactions were performed in duplicate in the *NOTCH1* gene in a 15 μl final volume using a Type-it HRM PCR Kit (Qiagen, Hilden, Germany). A 1 × HRM PCR master mix contained HotStar Taq Plus DNA polymerase, Type-it HRM PCR buffer, Q-solution, dNTP and EVA green dye, 15 ng DNA, and 0.66 μM of each primer was prepared. HRM assays were conducted with LightCycler® 480 Instrument (Roche Diagnostics) and LightCycler® 480 Gene Scanning Software Ver. 1.5 (Roche Diagnostics) for analysis. With SYBR Green I filter (533 nm), the PCR programme consisted of an initial denaturation-activation step at 95 °C for 10 min and a 40-cycle programme for detecting the *NOTCH1* gene (denaturation at 95 °C for 10s, annealing at 63 °C 35s, and elongation at 72 °C for 10s) to read the fluorescence in single acquisition mode. The melting programme included denaturing at 95 °C for 1 min, annealing at 40 °C for 1 min, and subsequent melting that involved a continuous fluorescent reading of fluorescence from 55 to 90 °C at the rate of 25 acquisitions per °C. The curve plotted for each DNA duplicate sample was reproducible in terms of both shape and peak height. To verify the results of HRM analysis, Sanger DNA sequencing analysis was performed for all the amplicons containing an abnormal melting curve and some of the amplicons with a normal melting curve (Table S2).

### Genotyping of NOTCH1 SMs-related SNPs

Based on *NOTCH1* SMs discovery, the genetic SNPs closest to SMs linkage disequilibrium (LD) >0.9 and allele frequencies >1% were included in a case-control study. Only one potential SNP (rs139994842) was genotyped using Sequenom MassARRAY System (San Diego, CA) at the Academia Sinica National Genotyping Center (Taipei, Taiwan).

### *In silico* prediction of NOTCH1 SMs in EGF-like domains

Fig. S4 shows a three-dimensional (3D) protein structure to provide insight into protein function. The crystal structure of EGF11-13 repeats (PDB ID: 2VJ3) include the ligand binding site and an almost linear domain arrangement^38^. The *O*-glycan is observed in an interaction between the disaccharide in the *NOTCH1* and protein side chains in its ligand using the 3D structures of PDB ID 4XL1^39^ in the Ca^2+^ stabilized EGF-like domains and the NMR structure PDB ID: 1TOZ^40^.

### Statistical analysis

Clinical characteristics were analysed using a Chi-square test. The odd ratios of cancer recurrence and death, unadjusted or adjusted for surgery age, disease status (site and stage of cancer), or adjuvant therapies (radiotherapy and chemotherapy), were calculated using logistic regression models. The Kaplan–Meier estimated a 5-year and 10-year relapse-free survival and recurrence rate. Differences in recurrence and survival proportions between patients detected with and without *NOTCH1* SMs were tested by a log-rank test. A multivariate Cox proportional-hazards regression analysis evaluated the prognostic factor of *NOTCH1* SMs associated with recurrence and survival of HNSCC patients. Clinical factors (age at surgery, cancer site, cancer stage, radiotherapy and chemotherapy) were analysed as potential covariates in models. To identify which germline genetic variant has contributed to a detectable SM, a logistic regression analysis was performed to estimate the association between germline variant and SMs. All tests are two-tailed and a *P* value < 0.05 is considered to be statistically significant.

## Additional Information

**How to cite this article**: Liu, Y.-F. *et al.* Somatic Mutations and Genetic Variants of NOTCH1 in Head and Neck Squamous Cell Carcinoma Occurrence and Development. *Sci. Rep.* **6**, 24014; doi: 10.1038/srep24014 (2016).

## Supplementary Material

Supplementary Information

## Figures and Tables

**Table 1:** Clinical characteristics of HNSCC patients in a 13 years of follow-up cohort.

Characteristics	HNSCC male patients (n = 128)		*P* value
	Without SMs (n = 105)	With SMs (n = 23)	
Age (years, mean ± SD)	51.3 ± 10.6	52.8 ± 12.2	0.56
Primary cancer site			
Buccal	7 (30.4%)	48 (45.7%)	0.83
Tongue	9 (39.1%)	33 (31.4%)	
Gum	3 (12.0%)	12 (11.4%)	
Palate	2 (8.7%)	5 (4.8%)	
Lip	1 (4.3%)	2 (1.9%)	
Oropharynx	1 (4.3%)	2 (1.9%)	
Hypopharynx	0 (0.0%)	1 (1.0%)	
Larynx	0 (0.0%)	1 (1.0%)	
Stage			
I	4 (17.4%)	15 (14.3%)	0.79
II	3 (13.0%)	24 (22.9%)	
III	6 (26.1%)	27 (25.7%)	
IV	9 (39.1%)	38 (36.2%)	
Radiotherapy			
Yes	14 (60.9%)	71 (67.6%)	0.51
No	8 (34.8%)	27 (25.7%)	
Chemotherapy			
Yes	11 (47.8%)	58 (55.2%)	0.28
No	12 (52.2%)	43 (41.0%)	
Recurrence			
Yes	12 (52.2%)	27 (25.7%)	0.02
No	11 (47.8%)	78 (74.3%)	
Fatality			
Yes	7 (30.4%)	15 (14.3%)	0.06
No	16 (69.6%)	90 (85.7%)	

**Table 2:** Hazard ratio (HR) of HNSCC recurrence and fatality during 5-year relapse-free follow-up.

Endpoint	HNSCC male patients (n = 128)			*P* value
	Without SMs (n = 105)	With SMs (n = 23)		
Recurrence				
HR, 95% CI	1.0	3.2	(1.5–6.6)	<0.01
HR, 95% CI†	1.0	3.5	(1.6–7.5)	<0.01
HR, 95% CI‡	1.0	5.2	(2.1–12.5)	<0.01
Fatality				
HR, 95% CI	1.0	2.5	(1.0–6.0)	0.05
HR, 95% CI†	1.0	2.7	(1.1–7.0)	0.04
HR, 95% CI‡	1.0	5.2	(1.6–16.8)	<0.01

^†^Model 1: OR was adjusted for surgery, age, cancer and stage.

^‡^Model 2: OR was adjusted for surgery, age, cancer, stage, radiotherapy and chemotherapy.

**Table 3:** NOTCH1 genetic variant (rs139994842) linked to somatic mutations in NOTCH1 is associated with betel quid and HNSCC occurrence using logistic regression adjusted age and substances use covariates.

Subjects	NOTCH1 rs139994842						Alcohol drinking				Betel quid chewing				Cigarette smoking			
	A/G	G/G	r	D’	OR (95% CI)	*p*-value	Yes	No	OR (95% CI)	*p*-value	Yes	No	OR (95% CI)	*p*-value	Yes	No	OR (95% CI)	*p*-value
Case-control study group (282 patients and 282 controls)																		
Control	8	274			1.00		82	200	1.00		39	243	1.00		149	133	1.00	
HNSCC	17	264			2.34 (1.02–5.46)*	0.04	190	92	1.46 (0.88–2.42)	0.21	232	50	22.45 (13.39–37.64)	<.0001	243	39	1.34 (0.77–2.34)	0.24
					3.46 (1.11–10.84)**	0.03												
SMs validation group (128 patients)																		
Without SMs	5	99			1.73 (0.55–5.41)*	0.37	87	16	5.71 (2.39–13.64)	0.0013	85	8	31.55 (13.00–76.60)	<.0001	84	21	0.98 (0.38–2.54)	0.25
					1.15 (0.24–5.50)**	0.85												
With SMs	5	19	0.63	1	9.01 (2.69–30.23)*	0.0003	18	5	2.17 (0.59- 8.03)	0.35	20	3	32.11 (10.41–99.05)	<.0001	18	5	0.36 (0.05–2.36)	0.13
					7.74 (1.70–35.31)**	0.008												

r: Correlation coefficient between polymorphisms and somatic mutation (*P* value = 0.0004).

D’: The coefficient of linkage disequilibrium between SMs and rs139994842.

^*^Adjusted for substances use.

^**^Adjusted for substances use and age.

## References

[b1] Ferlay J. *et al.* Estimates of worldwide burden of cancer in 2008: GLOBOCAN 2008. International journal of cancer. Journal international du cancer 127, 2893–2917 (2010). 21351269 10.1002/ijc.25516

[b2] Ferlay J. *et al.* Cancer incidence and mortality worldwide: sources, methods and major patterns in GLOBOCAN 2012. International journal of cancer. Journal international du cancer 136, E359–386 (2015). 25220842 10.1002/ijc.29210

[b3] Sharan R. N., Mehrotra R., Choudhury Y. & Asotra K. Association of betel nut with carcinogenesis: revisit with a clinical perspective. PLos One 7, e42759 (2012). 22912735 10.1371/journal.pone.0042759 PMC3418282

[b4] Cancer Genome Atlas, N. Comprehensive genomic characterization of head and neck squamous cell carcinomas. Nature 517, 576–582 (2015). 25631445 10.1038/nature14129 PMC4311405

[b5] Agrawal N. *et al.* Exome sequencing of head and neck squamous cell carcinoma reveals inactivating mutations in NOTCH1. Science 333, 1154–1157 (2011). 21798897 10.1126/science.1206923 PMC3162986

[b6] Stransky N. *et al.* The mutational landscape of head and neck squamous cell carcinoma. Science 333, 1157–1160 (2011). 21798893 10.1126/science.1208130 PMC3415217

[b7] Chillakuri C. R., Sheppard D., Lea S. M. & Handford P. A. Notch receptor-ligand binding and activation: insights from molecular studies. Seminars in cell & developmental biology 23, 421–428 (2012). 22326375 10.1016/j.semcdb.2012.01.009 PMC3415683

[b8] Sakamoto K. *et al.* Reduction of NOTCH1 expression pertains to maturation abnormalities of keratinocytes in squamous neoplasms. Laboratory investigation; a journal of technical methods and pathology 92, 688–702 (2012). 10.1038/labinvest.2012.9 22330335

[b9] Feigelson H. S. *et al.* Approaches to integrating germline and tumor genomic data in cancer research. Carcinogenesis 35, 2157–2163 (2014). 25115441 10.1093/carcin/bgu165 PMC4178473

[b10] Hsieh L. L. *et al.* Characteristics of mutations in the p53 gene in oral squamous cell carcinoma associated with betel quid chewing and cigarette smoking in Taiwanese. Carcinogenesis 22, 1497–1503 (2001). 11532872 10.1093/carcin/22.9.1497

[b11] Lobry C., Oh P. & Aifantis I. Oncogenic and tumor suppressor functions of Notch in cancer: it’s NOTCH what you think. The Journal of experimental medicine 208, 1931–1935 (2011). 21948802 10.1084/jem.20111855 PMC3182047

[b12] IARC. Betel-quid and areca-nut chewing and some areca-nut derived nitrosamines. IARC Monogr Eval Carcinog Risks Hum 85, 1–334 (2004). 15635762 PMC4781453

[b13] Ko Y. C. *et al.* Betel quid chewing, cigarette smoking and alcohol consumption related to oral cancer in Taiwan. J Oral Pathol Med 24, 450–453 (1995). 8600280 10.1111/j.1600-0714.1995.tb01132.x

[b14] Stewart B. W. & Kleihues P. World Cancer Report 2003. http://www.iarc.fr/en/publications/pdfs-online/wcr/2003/ (2003), (Data of access:14/12/2015).

[b15] Buch S. C., Notani P. N. & Bhisey R. A. Polymorphism at GSTM1, GSTM3 and GSTT1 gene loci and susceptibility to oral cancer in an Indian population. Carcinogenesis 23, 803–807 (2002). 12016153 10.1093/carcin/23.5.803

[b16] Hiruma-Shimizu K. *et al.* Chemical synthesis, folding, and structural insights into O-fucosylated epidermal growth factor-like repeat 12 of mouse Notch-1 receptor. J Am Chem Soc 132, 14857–14865 (2010). 20883017 10.1021/ja105216u

[b17] Teodorczyk M. & Schmidt M. H. Notching on Cancer’s Door: Notch Signaling in Brain Tumors. Front Oncol 4, 341 (2014). 25601901 10.3389/fonc.2014.00341 PMC4283135

[b18] Chiang S. L. *et al.* Up-regulation of inflammatory signalings by areca nut extract and role of cyclooxygenase-2 -1195G > a polymorphism reveal risk of oral cancer. Cancer research 68, 8489–8498 (2008). 18922923 10.1158/0008-5472.CAN-08-0823

[b19] Lee K. W. *et al.* Different impact from betel quid, alcohol and cigarette: risk factors for pharyngeal and laryngeal cancer. Int J Cancer 117, 831–836 (2005). 15957167 10.1002/ijc.21237

[b20] Lee C. H. *et al.* The neoplastic impact of tobacco-free betel-quid on the histological type and the anatomical site of aerodigestive tract cancers. International journal of cancer. Journal international du cancer 131, E733–743 (2012). 22174014 10.1002/ijc.27401

[b21] Humans, I.W.G.o.t.E.o.C.R.t. Betel-quid and areca-nut chewing and some areca-nut derived nitrosamines. IARC Monogr Eval Carcinog Risks Hum 85, 1–334 (2004). 15635762 PMC4781453

[b22] Boucher B. J. & Mannan N. Metabolic effects of the consumption of Areca catechu. Addict Biol 7, 103–110 (2002). 11900629 10.1080/13556210120091464

[b23] Song Y. *et al.* Identification of genomic alterations in oesophageal squamous cell cancer. Nature 509, 91–95 (2014). 24670651 10.1038/nature13176

[b24] Sun W. *et al.* Activation of the NOTCH pathway in head and neck cancer. Cancer research 74, 1091–1104 (2014). 24351288 10.1158/0008-5472.CAN-13-1259 PMC3944644

[b25] Turner E. H., Ng S. B., Nickerson D. A. & Shendure J. Methods for genomic partitioning. Annu Rev Genomics Hum Genet 10, 263–284 (2009). 19630561 10.1146/annurev-genom-082908-150112

[b26] Li H. & Durbin R. Fast and accurate short read alignment with Burrows-Wheeler transform. Bioinformatics 25, 1754–1760 (2009). 19451168 10.1093/bioinformatics/btp324 PMC2705234

[b27] Langmead B. & Salzberg S. L. Fast gapped-read alignment with Bowtie 2. Nature methods 9, 357–359 (2012). 22388286 10.1038/nmeth.1923 PMC3322381

[b28] Ewing A. D. *et al.* Combining tumor genome simulation with crowdsourcing to benchmark somatic single-nucleotide-variant detection. Nature methods 12, 623–630 (2015). 25984700 10.1038/nmeth.3407 PMC4856034

[b29] Danecek P. *et al.* The variant call format and VCFtools. Bioinformatics 27, 2156–2158 (2011). 21653522 10.1093/bioinformatics/btr330 PMC3137218

[b30] Cibulskis K. *et al.* Sensitive detection of somatic point mutations in impure and heterogeneous cancer samples. Nature biotechnology 31, 213–219 (2013). 10.1038/nbt.2514 PMC3833702 23396013

[b31] Larson D. E. *et al.* SomaticSniper: identification of somatic point mutations in whole genome sequencing data. Bioinformatics 28, 311–317 (2012). 22155872 10.1093/bioinformatics/btr665 PMC3268238

[b32] Wang K., Li M. & Hakonarson H. ANNOVAR: functional annotation of genetic variants from high-throughput sequencing data. Nucleic acids research 38, e164 (2010). 20601685 10.1093/nar/gkq603 PMC2938201

[b33] Genomes Project C. *et al.* An integrated map of genetic variation from 1,092 human genomes. Nature 491, 56–65 (2012). 23128226 10.1038/nature11632 PMC3498066

[b34] Sherry S. T. *et al.* dbSNP: the NCBI database of genetic variation. Nucleic acids research 29, 308–311 (2001). 11125122 10.1093/nar/29.1.308 PMC29783

[b35] Minton J. A., Flanagan S. E. & Ellard S. Mutation surveyor: software for DNA sequence analysis. Methods in molecular biology 688, 143–153 (2011). 20938837 10.1007/978-1-60761-947-5_10

[b36] Forbes S. A. *et al.* COSMIC: exploring the world’s knowledge of somatic mutations in human cancer. Nucleic acids research 43, D805–D811 (2015). 25355519 10.1093/nar/gku1075 PMC4383913

[b37] Er T. K. & Chang J. G. High-resolution melting: applications in genetic disorders. Clinica chimica acta; international journal of clinical chemistry 414, 197–201 (2012). 10.1016/j.cca.2012.09.012 22995429

[b38] Cordle J. *et al.* A conserved face of the Jagged/Serrate DSL domain is involved in Notch trans-activation and cis-inhibition. Nature structural & molecular biology 15, 849–857 (2008). 10.1038/nsmb.1457 PMC2669539 18660822

[b39] Luca V. C. *et al.* Structural biology. Structural basis for Notch1 engagement of Delta-like 4. Science 347, 847–853 (2015). 25700513 10.1126/science.1261093 PMC4445638

[b40] Hambleton S. *et al.* Structural and functional properties of the human notch-1 ligand binding region. Structure 12, 2173–2183 (2004). 15576031 10.1016/j.str.2004.09.012

