# Corrigendum: Somatic Mutations and Genetic Variants of NOTCH1 in Head and Neck Squamous Cell Carcinoma Occurrence and Development

**DOI:** 10.1038/srep28409

**Published:** 2016-07-11

**Authors:** Yu-Fan Liu, Shang-Lun Chiang, Chien-Yu Lin, Jan-Gowth Chang, Chia-Min Chung, Albert Min-Shan Ko, You-Zhe Lin, Chien-Hung Lee, Ka-Wo Lee, Mu-Kuan Chen, Chun-Hung Hua, Ming-Hsui Tsai, Yuan-Chien Chen, Ying-Chin Ko

Scientific Reports 6: Article number: 2401410.1038/srep24014; published online: 04012016; updated: 07112016

This Article contains errors in Table 1, where the column headings ‘Without SMs (n = 105)’ and ‘With SMs (n = 23) are inverted. The correct Table 1 appears below.

## Figures and Tables

**Table 1:** Clinical characteristics of HNSCC patients in a 13 years of follow-up cohort.

Characteristics	HNSCC male patients (n = 128)		P value
	With SMs (n = 23)	Without SMs (n = 105)	
Age (years, mean ± SD)	52.8 ± 12.2	51.3 ± 10.6	0.56
Primary cancer site			
Buccal	7 (30.4%)	48 (45.7%)	0.83
Tongue	9 (39.1%)	33 (31.4%)	
Gum	3 (12.0%)	12 (11.4%)	
Palate	2 (8.7%)	5 (4.8%)	
Lip	1 (4.3%)	2 (1.9%)	
Oropharynx	1 (4.3%)	2 (1.9%)	
Hypopharynx	0 (0.0%)	1 (1.0%)	
Larynx	0 (0.0%)	1 (1.0%)	
Stage			
I	4 (17.4%)	15 (14.3%)	0.79
II	3 (13.0%)	24 (22.9%)	
III	6 (26.1%)	27 (25.7%)	
IV	9 (39.1%)	38 (36.2%)	
Radiotherapy			
Yes	14 (60.9%)	71 (67.6%)	0.51
No	8 (34.8%)	27 (25.7%)	
Chemotherapy			
Yes	11 (47.8%)	58 (55.2%)	0.28
No	12 (52.2%)	43 (41.0%)	
Recurrence			
Yes	12 (52.2%)	27 (25.7%)	0.02
No	11 (47.8%)	78 (74.3%)	
Fatality			
Yes	7 (30.4%)	15 (14.3%)	0.06
No	16 (69.6%)	90 (85.7%)	

