# Supplementary material for: Somatic Mutations and Genetic Variants of NOTCH1 in Head and Neck Squamous Cell Carcinoma Occurrence and Development
Source: Sci Rep. 2016 Apr 1;6:24014. doi: 10.1038/srep24014 (PMC4817511; doi:10.1038/srep24014)
Supplement: Supplementary Information [file srep24014-s1.pdf]

Title

Somatic Mutations and Genetic Variants of NOTCH1 in Head and Neck Squamous Cell Carcinoma Occurrence and Development

Author list and affiliations

Yu-Fan Liu[1,2#], Shang-Lun Chiang[3,4#], Chien-Yu Lin[5,12], Jan-Gowth Chang[5], Chia-Min Chung[3], Albert Min-Shan Ko[6], You-Zhe Lin[3], Chien-Hung Lee[6], Ka-Wo Lee[8], Mu-Kuan Chen[9], Chun-Hung Hua[10], Ming-Hsui Tsai[10], Yuan-Chien Chen[11], Ying-Chin Ko[3,12]*

1. Department of Biomedical Sciences, College of Medicine Sciences and Technology, Chung Shan Medical University, Taichung, Taiwan
2. Division of Allergy, Department of Pediatrics, Chung-Shan Medical University Hospital, Taichung, Taiwan
3. Environment-Omics-Diseases Research Centre, China Medical University Hospital, Taichung, Taiwan
4. Department of Health Risk Management, College of Public Health, China Medical University, Taichung, Taiwan
5. Department of Laboratory Medicine, China Medical University Hospital, Taichung, Taiwan
6. Department of Evolutionary Genetics, Max Planck Institute for Evolutionary Anthropology, Leipzig D-04103, Germany
7. Department of Public Health, College of Health Science, Kaohsiung Medical University, Taiwan
8. Department of Otolaryngology, Kaohsiung Medical University Hospital, Kaohsiung, Taiwan
9. Oral Cancer Center, Changhua Christian Hospital, Changhua, Taiwan
10. Department of Otorhinolaryngology, China Medical University Hospital, Taichung, Taiwan
11. Department of Dentistry, China Medical University Hospital, Taichung, Taiwan
12. Graduate Institute of Clinical Medical Science, China Medical University, Taichung, Taiwan

#Dr. Liu and Dr. Chiang contributed equally to this work.

***Correspondence to**: Ying-Chin Ko, MD, PhD, Professor, Graduate Institute of Clinical Medical Science, China Medical University, 2 Yude Road, Taichung, 40447, Taiwan. TEL 886-4-2205-2121 ext.7621.

E-mail: ycko0406@gmail.com, ycko@mail.cmu.edu.tw.

Figure S1

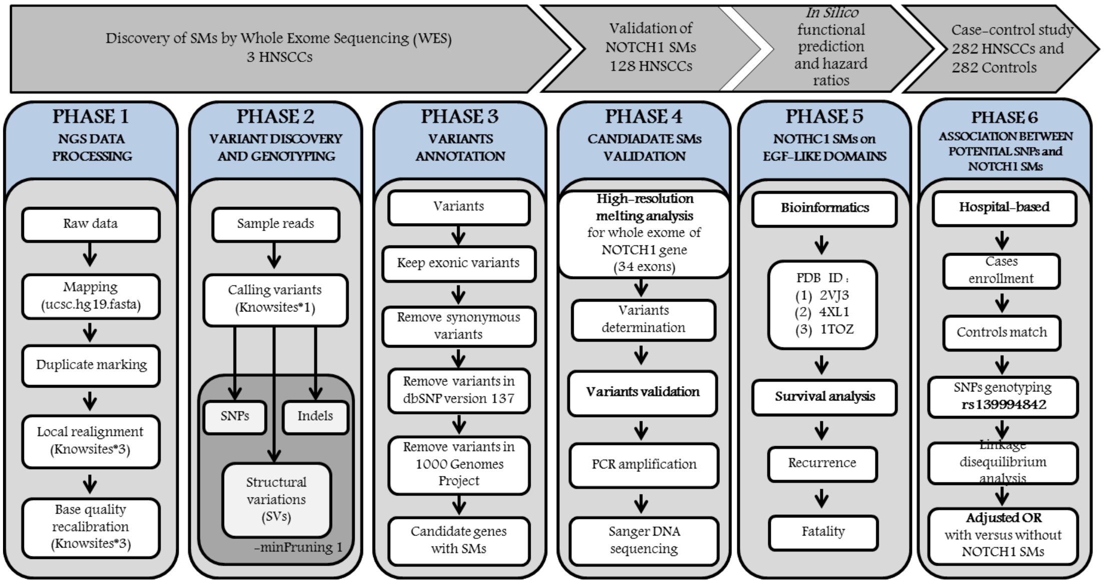

Figure S1 | Overview of approach to determine the somatic mutations and genetic variants in this study. Phase 1 to 3 are whole-exome sequencing (WES) discovery (first cohort: paired tissues from 3 patients), Phase 4 is validation using High-resolution melting (HRM) and Sanger sequencing (second cohort: paired tissues from 128 patients), Phase 5 is *in silico* functional prediction and hazard ratio statistic methods. Phase 6 is a case-control association study of SNPs and somatic mutations in investigation of substance use (third cohort: 282 patients and 282 controls).

Figure S2

Symbol	chr	start	end	ref	alt	AAChange.refGene	Func.refGene
NOTCH1	chr9	139413214	139413214	C	T	NOTCH1:NM_017617:exon6: c.G928A:p.G310R	nonsynonymous SNV

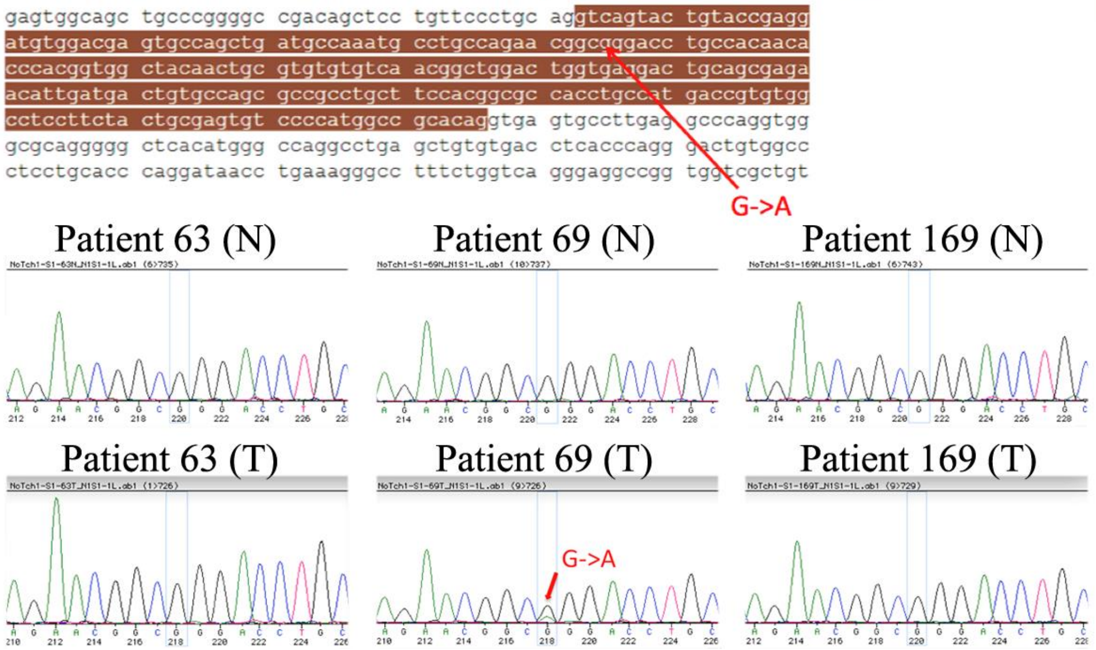

Figure S2 | Validation of the somatic mutations from NGS screening. NOTCH1 SMs detected from paired tissues (cancerous and marginal normal parts) were validated using Sanger DNA sequencing through forward and reverse direction, respectively, for 3 HNSCC patients.

Figure S3

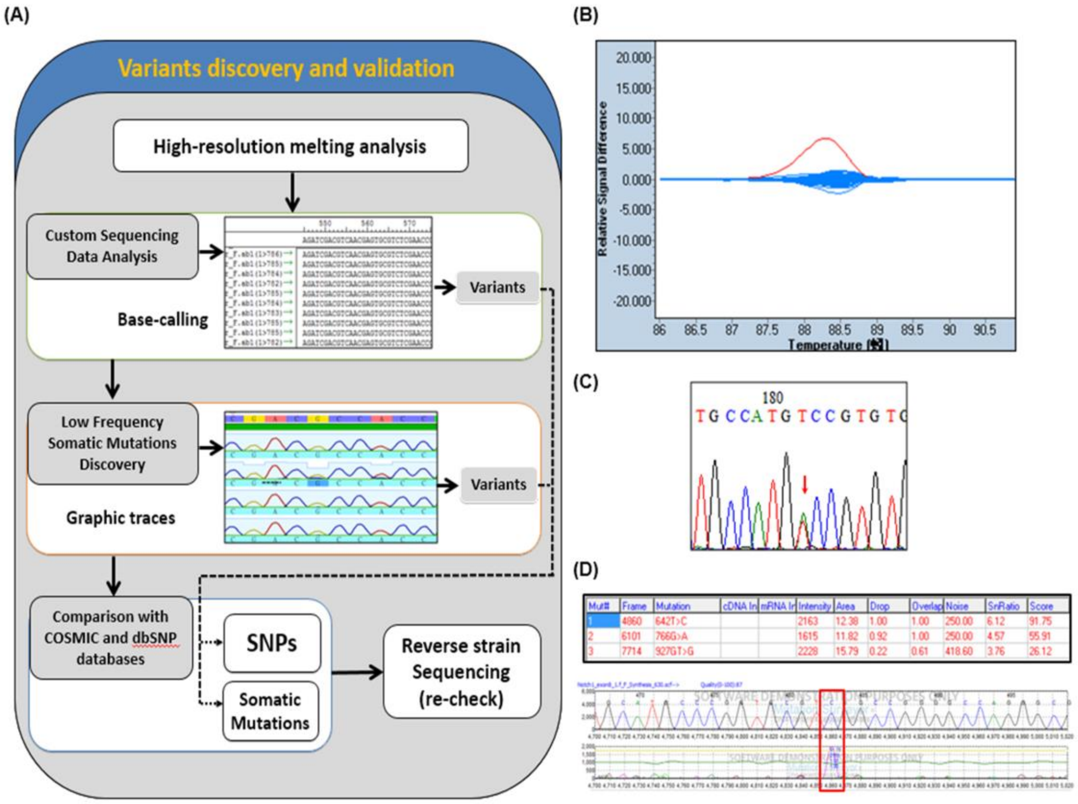

Figure S3 | Detection of somatic mutations from 128 HNSCC patients using high-resolution melting analysis and Sanger resequencing. (A) All the hotspot mutations identified in the discovery of variants using the HRM to high-throughput detection in the targeted sequences were validated by Sanger sequencing the tumour and the matched normal clinical DNA. The sequence graphs were analysed with the Mutation Surveyor software (version 4.0.6, Softgenetics^{TM}, State College, PA) **(B)** The HRM analysis melting profile gives a specific sequence-related pattern allowing discrimination between wild-type (blue line) sequences and homozygote-heterozygote variants (red line). HRM analysis offers a faster and more convenient closed-tube method of assessing the presence of variants and gives a result that can be further investigated if it is of interest. **(C)** A case of a single, unmatched base calling of a true variant. Additionally, in this sample, the variant score reached a set threshold value. **(D)** Output table of a heterozygous analysed sequence data from the electro-photogram was the variant photogram which highlights the red box as the differences between normal and reference traces as spikes. Scores are derived from the signal to noise ratio with the dropping factor and overlapping factor expressed as -10 log (error probability).

Figure S4

(A)

(B)

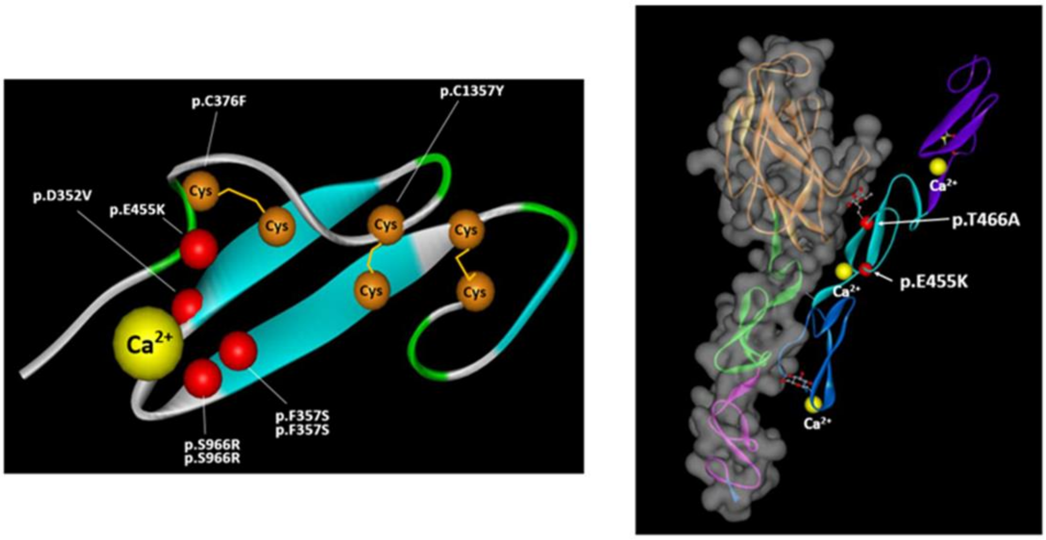

Figure S4 | Eight missense somatic mutations mapping onto the structural model of the ligand interaction and Ca^{2+}-binding EGF-like domain of NOTCH1 in this study. (A) The ribbon diagram was generated with Viewerlite 5.0 showing the 3D structure (PDB accession number: 2VJ3) of the Ca^{2+}-binding EGF-like domain of NOTCH1. A space-filling representation is shown around the central Ca^{2+}. There are five side-chain oxygen ligands in an approximately pentagonal arrangement coordination of Ca^{2+}. The disulfide bonds of six conserved oxygen ligands are shown in the present by stick. The red sphere indicated the Cα carbon of the somatic mutations characterized in the study. **(B)** The location and substitutions responsible for the ligand interaction somatic mutations (red spheres of the wild-type C-alpha atoms) are clustered at the interface between the EGF-like domains of NOTCH1 and ligand binding domain of DLL4 indicated on a three-dimensional model (PDB accession number: 4XL1). Single letter abbreviation of amino acid is used, and the NOTCH1 is displayed as a backbone cartoon (coloured with blue, cyan and purple indicated the EGF-like domain 11, 12 and 13, respectively) complex with space filling representations of its ligand DLL4 (coloured with pink, green and orange revealed the EGF1, DSL and MNNL, respectively). The white surface of DLL4 represents the contact interface with NOTCH1. Ca^{2+} ions are shown as yellow spheres, and NOTCH1 *O*-glycans are highlighted in ball-and-stick mode.

Figure S5

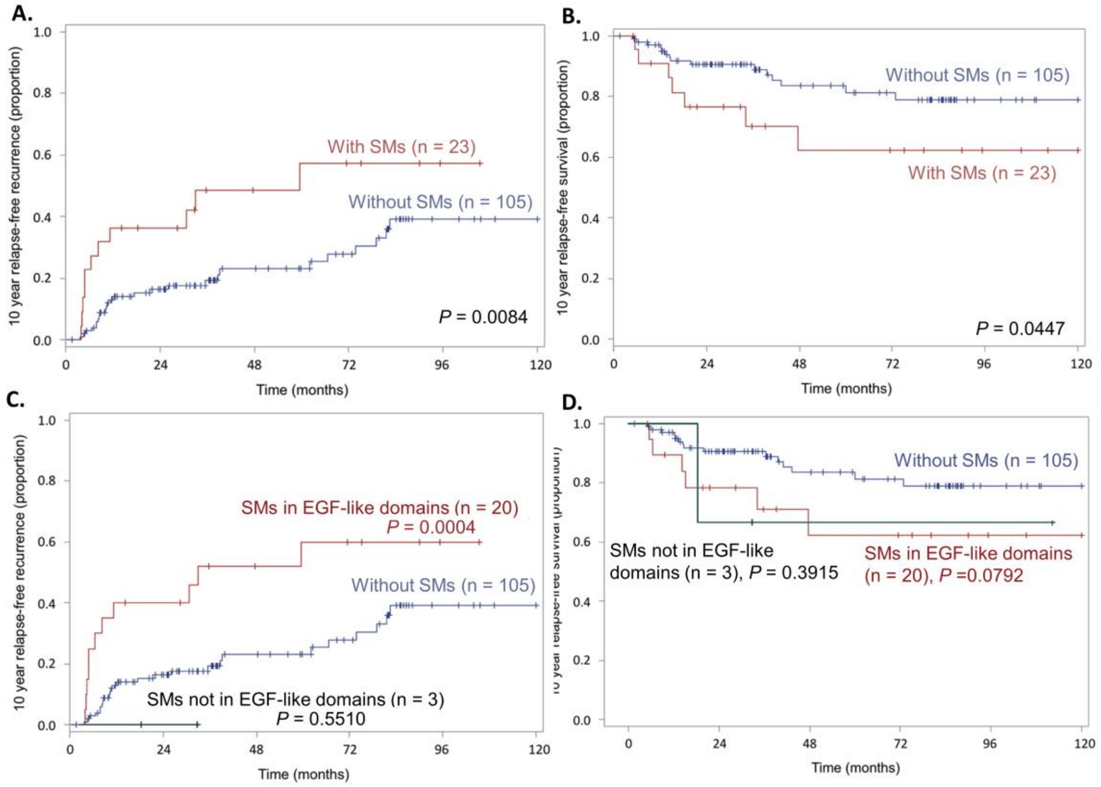

Figure S5 | Kaplan-Meier estimates of 10-year relapse-free recurrence and survival proportion in 128 HNSCC patients. (A) Patients with SMs have significantly higher recurrence rate, **(B)** Patients with SMs have significantly lower survival rate, **(C)** Patients with SMs in EGF-like domain (representing majority of SMs found in this study) have significantly higher recurrence rate, **(D)** Patients with SMs in EGF-like domains vs. not in EGF-like domains may have lower survival rate (*P*=0.0792 vs. *P*=0.3915).

Table S1 | SMs identified in NOTCH1 gene from 23 HNSCC patients.

#	Case ID [a]	Exon	Genome Positions [b]	cDNA position [c]	AA Change	Variant type	Effected domains [d]	Predicted Effect	COSMIC ID [f]
1	X06216001	4	139,417,387	c.657C>T	p.Y219	synonymous	-	-	COSM1106904
2	X06023402	6	139,413,161	c.981G>C	p.W327C	missense	EGF-like repeats	-	-
3	T11369702	6	139,413,087	c.1055A>T	p.D352V	missense	EGF-like repeats	Calcium binding	-
4	X07308202	6	139,413,072	c.1070T>C	p.F357S	missense	EGF-like repeats	Calcium binding	COSM4984793
5	X07325401	6	139,413,072	c.1070T>C	p.F357S	missense	EGF-like repeats	Calcium binding	COSM4984793
6	X05184701	7	139,412,717	c.1127G>T	p.C376F	missense	EGF-like repeats	Disulfide binds	-
7	X08162002	7	139,412,690	c.1154C>A	p.S385Y	missense	EGF-like repeats	*O*-glucosylation site	-
8	X06125403	7	139,412,661	c.1184A>T	p.K395X	nonsense	EGF-like repeats	Truncated form	-
9	T11042802	8	139,412,282	c.1363G>A	p.E455K	missense	EGF-like repeats	Calcium binding and Ligand binding site	COSM4525934
10	X06065401	8	139,412,249	c.1396A>G	p.T466A	missense	EGF-like repeats	*O*-glucosylation site and Ligand binding site	-
11	X05030902	12	139,409,770	c.1986C>G	p.Y662X	nonsense	EGF-like repeats	Truncated form	-
12	T09269701	12	139,409,752	c.2004insC	-	frameshift	EGF-like repeats	Truncated form	-
13	X08049403	13	139,408,989	c.2180A>T	p.H727L	missense	EGF-like repeats	Truncated form	-
14	X06125403	17	139,405,191	c.2654insC	-	frameshift	EGF-like repeats	Truncated form	-
15	T11104401	17	139,405,192	c.2650_2653delTGCC	-	frameshift	EGF-like repeats	Truncated form	-
16	T06160601	18	139,404,256	c.2898C>G	p.S966R	missense	EGF-like repeats [e]	Calcium binding	-
17	X06160602	18	139,404,256	c.2898C>G	p.S966R	missense	EGF-like repeats [e]	Calcium binding	-
18	X06200503	20	139,402,687	c.3322C>T	p.Q1108X	nonsense	EGF-like repeats [e]	Truncated form	-
19	X04179501	22	139,401,813	c.3587G>A	p.G1196D	missense	EGF-like repeats	-	COSM4544496
20	T11398301	22	139,401,758	c.3642G>A	p.Q1214	synonymous	-	-	-
21	X08166602	25	139,400,278	c.4070G>A	p.C1357Y	missense	EGF-like repeats	Disulfide binds	-
22	T11159201	25	139,400,017	c.4331T>A	p.L1444Q	missense	LNR	-	-
23	X05123402	28	139,396,889	c.5119C>T	p.A1740V	missense	Transmembrane region	Transmembrane region	-
24	T11291901	30	139,396,302	c.5536C>T	p.Q1846X	nonsense	RAM	Truncated form	-

a. Totally, 124 patients were recruited to discovery and validate the NOTCH1 SMs in coding region. Twenty-three patients had NOTCH1 SMs, and patient X06125403 carried 2 different mutations.

b. All SMs coordinates were transferred to GRCH37.p13 (NC_000009.11) annotation release 105 and using human transcript annotation imported from Ensemble database release 69.

c. Nucleotides and amino acids numbers are according to the GenBank accession numbers NM_017617.3 and NP_060087.3, respectively.

d. The distribution of protein domains annotated by SMART database [2] including 36 tandem EGF repeats (amino acids 23 to 1,426), 3 Lin-12/N repeats (LNR, amino acids 1,442 to 1,562) and RBP-JK-associated molecule region (RAM, amino acids 1,757 to 1,865).

e. Abruptex : EGF repeats 24-29 is known for dominant mutation which activate Notch signaling bearing *O*-fucosylation sites.

f. Five somatic mutations were found in the database of Catalogue of Somatic Mutations in Cancer (COSMIC) v73, and 19 were novel in this study.

Table S2 | HRM analysis used the primers in the NOTCH1 coding region.

Primer name	Forward	Primer name	Reverse	Size (bp)
NOTCH1 E1-F	AATTTACGCCGCGCGTGT	NOTCH1 E1-R	CCAAAGGGCGCGGAAAGT	193
NOTCH1 E2-F	TGAGACTGACCTCTCTTCTCCTG	NOTCH1 E2-R	AAAGCAACAGGTCCCGCAG	145
NOTCH1 E3-F	TCTGGCACATCTGCCAACAG	NOTCH1 E3-R	AAGCTGTGGGTCCTCCCT	304
NOTCH1 E4-1-F	CTCTTGTCCCCTTGTCTCCAG	NOTCH1 E4-1-R	TAGGAGCCGACCTCGTTGTG	223
NOTCH1 E4-2-F	TCAACGAGTGTGGCCAGAAG	NOTCH1 E4-2-R	ACCAGCGGGCAGCACTAC	225
NOTCH1 E5-F	TACCTCAGGGAAGAGGCTGA	NOTCH1 E5-R	GTAAGTGGGTAGCAGCCCC	221
NOTCH1 E6-F	CAGCTCCTGTTCCCTGCAG	NOTCH1 E6-R	TGGGCCTCAAGGCACTCAC	272
NOTCH1 E7-F	TTTCCACTGACCGCCGCT	NOTCH1 E7-R	AACCTGTGCTGGCACCTAC	203
NOTCH1 E8-F	AAACAGCCTCTCACCCGTGT	NOTCH1 E8-R	CAGCCTCGACTCGGTTTCC	259
NOTCH1 E9-F	TCGTTTCTGTCCCAAGTCCAC	NOTCH1 E9-R	AAGCCAGGGTGCAGACGA	246
NOTCH1 E10-F	GAAGGGCCATAGTGCTGTTG	NOTCH1 E10-R	AGACCAAGGTGTCCATGACC	261
NOTCH1 E11-F	CCGCCAGTCCTAAGTCTTCC	NOTCH1 E11-R	ACGTGTCGGTCAGTCCTCA	348
NOTCH1 E12-F	TGAGGACTGACCGACACGT	NOTCH1 E12-R	TCTGAGCACAGTGCAGTCAG	186
NOTCH1 E13-F	TGGGCCTCGGAGTCTGAC	NOTCH1 E13-R	TGATGTGTCCCCATGATCGG	259
NOTCH1 E14-F	CTCGACCTGCAGTGTGGT	NOTCH1 E14-R	CACTGAGAAACGCGCAGC	201
NOTCH1 E15-F	GGACATGCCGAGTGCTGT	NOTCH1 E15-R	TAAAGCACAGGCCCCACC	174
NOTCH1 E16-F	TTACGTGTGTCTCCTTCCCG	NOTCH1 E16-R	ACAGACATCCTGACCTCCCA	196
NOTCH1 E17-F	TTGACCAACCGGCCTCCT	NOTCH1 E17-R	ATTCCTGGAGGAGGCCAGA	219
NOTCH1 E18-F	ACTCACCCTTCCGTCCTCT	NOTCH1 E18-R	TTCCACGGCCTCACTCGA	282
NOTCH1 E19-F	CACCAATGCCCTCCACTCA	NOTCH1 E19-R	TCCTTCGGGCACCTCTGT	280
NOTCH1 E20-F	CCAGCTGACCCCAATCTGT	NOTCH1 E20-R	GGTGGGCACAGCAGGTTAC	198
NOTCH1 E21-F	GGGCCTCACCTGTCTACCA	NOTCH1 E21-R	CGGCCACAACCCTTACCCTA	253
NOTCH1 E22-F	CGTTCTGTCGCCTGCACA	NOTCH1 E22-R	TACGTGCAGCGGCCCTTA	171
NOTCH1 E23-F	ACTGACGAAACCTGGCCC	NOTCH1 E23-R	TAACCCTGCTGCCCCACA	328
NOTCH1 E24-F	CTGCCCTGCTCTTACCCCTA	NOTCH1 E24-R	AATGCACCCCTGCACCTAC	153
NOTCH1 E25-1-F	ACCGTCCTGTCTTCCCTCT	NOTCH1 E25-1-R	TAGCACGCCTCCTCGATCA	361
NOTCH1 E25-2-F	TTGTGCCACATCCTGGACTAC	NOTCH1 E25-2-R	ATGAGCCCCGCAGCCTTA	336
NOTCH1 E26-1-F	TAAGGCGGCCTGAGCGT	NOTCH1 E26-1-R	AAGATCATCTGCTGGCCGT	297
NOTCH1 E26-2-F	AACGTGGTCTTCAAGCGTGA	NOTCH1 E26-2-R	GGAGAGTTGCGGGGATTGA	255
NOTCH1 E27-F	CTCTCTGATTGTCCGCCCAG	NOTCH1 E27-R	GGGATGGGGCCACACTTAC	188
NOTCH1 E28-F	TCTGATGTCCGGGCACCT	NOTCH1 E28-R	AACGGGGACCCAGAAGCA	294
NOTCH1 E29-F	GCTCAGCCTCACTTCTCGA	NOTCH1 E29-R	CATAGAGGGAGTGAGCAGAGC	206
NOTCH1 E30-F	GGCTCTGCTCACTCCCTCTA	NOTCH1 E30-R	GGCTGCTGGCACCCTTAC	209
NOTCH1 E31-F	AGACTGAGCACCCGTCTCT	NOTCH1 E31-R	ACAGGCAGCCACTGCCTA	344
NOTCH1 E32-F	TCCTGAGCCTCTCCCTGTT	NOTCH1 E32-R	AAACGACAGAGCAGCCGT	207
NOTCH1 E33-F	CCATCTGCTTCTTTCACGCAG	NOTCH1 E33-R	TGGATTCAGCCCTCACGTCT	166
NOTCH1 E34-F	CCTCTGGTGATGGAACCTTGG	NOTCH1 E34-R	CCGAAGGCTTGGGAAAGGAA	1,611

Table S3 | Characteristics of 564 HNSCC and controls were recruited to verify the association

Variable †	HNSCC N=282	Controls N=282	*P*-value
Male, N (%)	267 (94.7)	275(97.5)	0.08
Age, year (SD)	53.8 (10.4)	50.8 (14.3)	0.004
Alcohol, N (%)			
No drinker	92 (32.6)	200 (70.9)	< 0.0001
Drinkers	190 (67.4)	82 (29.1)	
Betel quid, N (%)			
No chewers	50 (17.7)	244 (86.5)	< 0.0001
Chewer	232 (82.3)	38 (13.5)	
Cigarette, N (%)			
No smokers	39 (13.8)	133 (47.2)	< 0.0001
Smokers	243 (86.2)	149 (52.8)	

† Substance users included ex-users and current users
